# Exacerbation induces a microbiota shift in sputa of COPD patients

**DOI:** 10.1371/journal.pone.0194355

**Published:** 2018-03-26

**Authors:** Eric Jubinville, Marc Veillette, Julie Milot, François Maltais, André M. Comeau, Roger C. Levesque, Caroline Duchaine

**Affiliations:** 1 Centre de recherche de l’Institut universitaire de cardiologie et de pneumologie de Québec (CRIUCPQ), Québec, Canada; 2 Département de biochimie, de microbiologie et de bio-informatique, Faculté des sciences et génie, Université Laval, Québec, Canada; 3 Département de médecine, Faculté de Médecine, Université Laval, Québec, Canada; 4 CGEB-Integrated Microbiome Resource (CGEB-IMR), Dalhousie University, Halifax, Canada; 5 Institut de Biologie Intégrative et des Systèmes (IBIS), Université Laval, Québec, Canada; Wageningen Universiteit, NETHERLANDS

## Abstract

Little is known about the microbiota shift induced by exacerbation in chronic obstructive pulmonary disease (COPD) patients. The sputa microbiota of COPD patients was evaluated when clinically stable and during acute exacerbations of the disease. Sputa microbiota was analyzed using 16S ribosomal RNA gene pyrosequencing and quantitative polymerase chain reaction-based pathogen detection. Nine COPD patients were enrolled. Pyrosequencing of 16S rRNA genes identified 2,267 unique bacterial operational taxonomic units. Principal microbiota shifts during exacerbation were in either *Proteobacteria*, *Firmicutes* or *Bacteroidetes*. *Streptococcus* and *Moraxella* levels were detected during exacerbation in severe (Global Initiative for Chronic Obstructive Lung Disease 3) COPD patients. Most of the clinically-important genera found in the sputum with the pyrosequencing of 16S rRNA gene correlated with specific quantitative polymerase chain reactions for bacteria while respiratory viruses were nearly absent. Sputum microbiotas of exacerbated COPD patients are complex. This pilot study shows a clear shift in the microbiota of patients during exacerbation. The nature of this shift varies from patient to patient in such a way that the treatment should be patient-specific. Further studies are needed to establish the impact of microbial exacerbations on the pulmonary microbiota.

## Introduction

Chronic obstructive pulmonary disease (COPD) is characterized by non-fully reversible airflow obstruction. It is currently the 4^th^ leading cause of death worldwide [1, 2], and has been projected by the World Health Organization to become the 3^rd^ by 2030 [3]. COPD patients often experience episodes of acute symptom worsening such as shortness of breath, wheezing, chronic cough, called acute exacerbations (AE) [4]. In fact, AE are an important cause of mortality and they are associated with the severity of the disease [5, 6].

Bacterial and/or viral infections are suspected to cause AE [7, 8]. *Haemophilus influenzae*, *Streptococcus pneumoniae*, *Pseudomonas aeruginosa* and *Moraxella catarrhalis* are frequently cultured from sputa of COPD patients during exacerbations [2, 9–12]. Human rhinovirus (HRV), Influenza A and B, and Respiratory syncytial virus (RSV) are viruses also detected in exacerbated COPD patients [13, 14].

In contrast to what was previously thought, recent microbiota studies have shown that the lungs are not sterile, even in healthy individuals [2, 15–17]. The lung microbiota of COPD patients has historically been investigated using culture-based methods with a limited view of biodiversity [11, 18–22]. Moreover, virus cultures are fastidious and have to be specifically adapted for the various virus types. Viral identification is also cumbersome using conventional culture-based methods [23].

Sze *et al*. characterized the microbiota of eight severe COPD patients using lung tissues and showed that *Proteobacteria* (44%), *Bacteroidetes* (28%), *Firmicutes* (16%), *Actinobacteria* (6%) and *Tenericutes* (5%) were the main phyla detected in those tissues. A significant increase of *Firmicutes* was shown compared to non-COPD patients included in their study (non-smokers, smokers and cystic fibrosis) [15]. Other studies suggested that *Firmicutes* [2, 24, 25], *Proteobacteria* [17] or both [26] are the main phyla found in the lungs of COPD patients. Results from these studies are, however, difficult to compare as the nature of the analyzed samples were different (bronchoalveolar lavage (BAL) or tissues). Importantly, the clinical use of these methods is limited by their invasive nature or their rarity (tissues). Sputum sampling is a rapid, low cost method, and non-invasive since most patients are able to spit spontaneously. However, sometimes saline is required in order to obtain induced sputum. Bronchoscopy with protected specimen brush (PSB) or bronchoalveolar lavage (BAL) require to locally disturb the lung tissue. To our knowledge, only one study compared the microbiota of stable and exacerbated patients using sputum analyses (n = 12) [27]. Huang et al. studied the dynamic (five samples/patient) of the lung microbiota and showed a predominance of *Proteobacteria* such as *Moraxellaceae*, *Pasteurellaceae*, *Pseudomonadaceae*, and *Enterobacteriaceae* at exacerbation in the sputum of COPD patients. They also pointed out that inhaled corticosteroids (ICs) may alter the microbiome, however viral analysis were not performed.

In this study, we used 16S rRNA gene pyrosequencing and quantitative PCR-based pathogen detection methods to characterize the sputum microbiota in patients with COPD during stable conditions and, subsequently, during acute COPD exacerbations (AECOPD), testing the hypothesis that there would be a shift sputum microbiotas during the exacerbation period. We also evaluated whether the sputum microbiotas in COPD are influenced by the severity of airflow limitation.

## Materials and methods

### Participants

Nine patients were recruited at the COPD clinic of the Institut Universitaire de cardiologie et de pneumologie de Québec (IUCPQ). Patients gave written consent and the study was approved by the IUCPQ’s Bureau du comité d’éthique de la recherche (BCÉR) (CÉR 2013–2115). Patients with chronic airflow limitation (forced expiratory volume in 1 s [FEV_1_]/forced vital capacity [FVC] < 0.7) were recruited based on a history of at least one exacerbation in the previous six months. Patients’ inclusion criteria were as follows: a cumulative smoking history ≥ 10 packs-year and a stable clinical condition with no antibiotics use for at least one month prior to collecting baseline sputum. Patients were then asked to contact the research team as soon as they were experiencing symptoms of clinical deterioration to reassess their sputum during an AECOPD. Patients were judged to have an AECOPD when two out of the three symptoms were seen (increased dyspnea, increased sputum volume and sputum color change (Anthonisen’s classification)) [28]. Patients on any antibiotic treatment, with infectious diseases such as HIV, tuberculosis, acute pneumonia and diagnosed with asthma were excluded from the study.

### Clinical data

Principal demographic data of each patient such as age, gender, Global Initiative for Chronic Obstructive Lung Disease (GOLD) classification and indices of lung function (FEV_1_, FVC, and FEV_1_/FVC) were collected.

### Sampling and sample processing

Sputa were collected from patients either spontaneously or by induction during stable condition and during the exacerbation period, before any use of antibiotics. Induced sputum was produced as follows: a saline solution (0.9%, 3.0%, 4.0% and 5.0%) was nebulised every seven minutes for a maximum of 21 minutes. Patients were asked to spit in a sterile tube.

Sputa were processed within two hours following collection. Briefly, mucus was selected from the specimen, suspended in phosphate buffer saline (1X PBS, pH 7.1; Cellgro, Manassas, Virginia, USA), treated with 0.2% dithiothreitol (DTT, EMD Millipore, Etobicoke, Ontario, Canada), weighed and frozen at -80°C until DNA and RNA extractions. All sputa samples passed the quality test (< 25 epithelial cells per low-power field).

### Strains and growth conditions

MS2 phage (HER-462) was obtained from the Félix d’Hérelle Reference Center for Bacterial Viruses (http://www.phage.ulaval.ca/). MS2 was cultured on *Escherichia coli* (from the American Type Culture Collection, ATCC-15597) at 37°C for 48 hours. *Pseudomonas aeruginosa* (ATCC-27853) was cultured in Tryptic Soy Broth (TSB; BD, Mississauga, Ontario, Canada) at 25°C for 48 hours. *Streptococcus pneumoniae* (HER-1054), *Moraxella catarrhalis* (ATCC-8176) and *Haemophilus influenzae* (ATCC-49247) were cultured at 37°C plus 5% CO_2_ on TSB for 72 hours, on blood agar (Oxoid Company, Nepean, Ontario, Canada) for 48 hours, or on chocolate agar (Oxoid Company) for 48 hours, respectively.

### DNA and RNA extraction

Sputum samples were completely thawed on ice, incubated with one equal volume of 10X DTT (100 mg = 100 μl DTT) at 37 °C for one hour, and vortexed every ten minutes [29]. Samples were then split in two for both DNA and RNA extractions. Prior to RNA extraction, samples were spiked with 1×10^7^ MS2 phages as an extraction and reverse transcription (RT) positive control. DNA and RNA were extracted with the MO BIO PowerLyzer DNA Extraction Kit (MOBIO, Carlsbad, California, USA) and AMBION MagMAX Viral RNA/DNA Isolation Kit (Life Technologies, Burlington, Ontario, Canada), respectively. RNA was eluted in 50 μL elution buffer heated at 56°C and immediately transformed into cDNA by RT. DNA was eluted in 50 μL and stored at -20°C prior to qPCR. DNA was quantified with a Thermo Scientific NanoDrop 2000 spectrophotometer (Thermo Scientific, Waltham, Massachusetts, USA).

### Reverse transcription

RT was performed with the iScript cDNA Kit (Bio-Rad, Mississauga, Ontario, Canada). Bio-Rad’s protocol was as follows: 5 minutes at 25°C, 30 minutes at 42°C and 5 minutes at 85°C. Each 40 μL reaction was composed of 30 μL of viral RNA template (limit of detection = <4 ng/μl), 8 μL of 5X iScript reaction mix and 2 μL of iScript reverse transcriptase. cDNA samples were stored at -20 °C prior to qPCR.

### qPCR standard curves

Genomic DNA extracted from *P*. *aeruginosa*, *M*. *catarrhalis*, and *H*. *influenzae* were used for standard curves for pathogen-specific qPCRs while *S*. *pneumoniae* genomic DNA was used for a standard curve for both total bacteria and *S*. *pneumoniae* qPCRs. Genomic DNA of these strains was extracted with the QIAGEN QIAmp DNA mini kit (QIAGEN, Toronto, Ontario, Canada).

Standard curves for viral pathogen quantification by qPCR were obtained from cloning cDNA of RSV A & B (RespiVir Study 2009–2010 #H0910-131 and ATCC VR-955 respectively), human rhinovirus (Rhinovirus-14) and adenovirus (ATCC VR-15) into pGC vectors according to manufacturer’s instructions (pGC Blue Cloning & Amplification Kit, Lucigen, Middleton, Wisconsin, USA). The viral cDNA was provided by the Centre Hospitalier Universitaire de Québec Pavillon CHUL (Québec, Québec, Canada).

### qPCR assays

Total, specific bacterial and viral pathogenic agents were quantified by qPCR. *P*. *aeruginosa*, *S*. *pneumoniae*, *M*. *catarrhalis*, *H*. *influenzae*, Influenza A and B, respiratory syncitial virus (RSV) A and B, adenovirus, and human rhinovirus were assessed using published primers and probes (S1 Table, [19, 30, 31]). All probes and primers were obtained from Integrated DNA Technologies (IDT, Coralville, Iowa, USA). Each qPCR reaction volume was composed of: 7.5 μL of Bio-Rad 2X iQ Supermix (2X reaction buffer with dNTPs, iTaq DNA polymerase, 6 mM MgCl_2_ and stabilizers), 1 μM forward primer, 1 μM reverse primer, 0.1 μM probe, 2 μL of DNA or cDNA template, and water (Sigma-Aldrich, St-Louis, Missouri, USA) up to the final volume of 15 μL. The thermal-protocol was as follows: 95°C for 3 min, followed by 40 cycles of 95°C for 10 s and 60°C for 30 s. All samples were analyzed in duplicate and water (Sigma) was used as a negative control. Reactions were manually loaded into Bio-Rad 96-well white plates. Amplifications were performed using a Bio-Rad CFX 96 Real-Time System and data acquired with the on-board CFX Manager Software version 3.0. qPCR efficiencies, detection limits and numbers of total bacteria and pathogenic agents were determined by employing serial 10-fold dilutions of the standard curves. All qPCR efficiencies were between 90–110%, with R^2^ values ranging between 0.96–0.99.

### 454 pyrosequencing of 16S rRNA genes

Universal primers were used to target the SSU rRNA gene within the V6-8 region of Bacteria [32]. Barcodes (TCB 2009–005) added were from the extended MID set (Roche, Branford, Connecticut, USA). PCR reactions contained: 1X Q5 buffer (New England Biolabs, Ipswich, Massachusetts, USA), 200 μM of each dNTP (Feldan, Quebec, Quebec, Canada), 0.2 μM of each 454 primer (IDT), 1 U of Q5 High-Fidelity DNA polymerase (NEB), and 1 μL of template DNA (10–50 ng). Cycling conditions were as follows: an initial denaturation at 98°C for 30 s, followed by 30 cycles of denaturation at 98°C for 10 s, annealing at 55°C for 30 s, extension at 72°C for 30 s, and a final extension at 72°C for 5 min. PCR products were purified using the Axyprep Mag PCR Clean-up Kit (Axygen, Union City, California, USA), and verified with a Bioanalyzer 2100 with DNA 7500 chips (Agilent Technologies, Santa Clara, California, USA). PCR products were then quantified with a NanoDrop 2000 spectrophotometer (Thermo Scientific). The 12 sample-coded amplicons were mixed in equal quantity and 1/8th plate was sequenced on a Roche 454 GS-FLX Titanium platform at the Plate-forme d’Analyses Génomiques de l’Université Laval (Québec, QC, Canada).

### Pre-processing and quality control of raw sequences

The datasets generated during the current study are available in the Sequence Read Archives (SRA) repository, identification number: SRP107187. Web link to the datasets: https://trace.ncbi.nlm.nih.gov/Traces/sra/sra.cgi?study=SRP107187. Raw reads were processed using the mothur pipeline (v1.30; http://www.mothur.org/) [33]. Low-quality reads were discarded: (1) containing one or more Ns, (2) short or long reads (<330 and >600 nt), (3) with incorrect forward primer sequence, and (4) homopolymers >8 nt. All nucleotides following the reverse primer were trimmed. Chloroplasts, mitochondria and putative chimeric reads were eliminated within mothur using included reference databases. Quality reads were aligned to the mothur-formatted SILVA reference [34]. The resulting alignments were then manually checked for misalignments and gaps using BioEdit v7.2.5 (Ibis Biosciences, Carlsbad, California, USA). Singletons were removed. Final quality reads, associated with a bar-code, were then randomly re-sampled in order to have the same number of sequences per sample (3,800).

### OTU and taxonomic analyses

Final quality reads were clustered into Operational Taxonomic Units (OTUs) at the 97% similarity level, which approximates genus for Bacteria [35, 36], using the furthest-neighbor clustering method in mothur, followed by calculations of diversity indices (Shannon and Simpson), rarefaction curves and community similarity values. Bacterial OTUs were taxonomically identified using the SILVA taxonomy outlines and reference sequence set provided with mothur (www.mothur.org/wiki/Silva_reference_files), trimmed to the V6-V8 region.

### Statistical analyses

Patients were categorized following the GOLD airflow limitation classification scheme which defines airflow limitation by and FEV_1_/FVC < 0.7 into GOLD 1 (FEV_1_ ≥ 80% predicted value), GOLD 2 (FEV_1_ 50–79% predicted value), GOLD 3 (FEV_1_ 30–49%) and GOLD 4 (FEV_1_ < 30% predicted value) [37]. Statistical analyses were performed using GraphPad Prism Software V. 6 (La Jolla, CA, USA). Baseline and exacerbated sputa microbiotas were compared using a paired patient t-test analysis (statistically significant when p ≤ 0.05). Comparisons between GOLD 3 and GOLD 2 COPD patients were performed with unpaired t-tests (statistically significant when p ≤ 0.05).

## Results

### Patient population

The severity of airflow limitation in the 9 recruited patients was as followed: GOLD 1, *n* = 1, GOLD 2, *n* = 3 and GOLD 3, *n* = 5. Patients’ characteristics are presented in Table 1.

**Table 1:** Demographic and clinical characteristics per patient.

	1	2	3	4	5	6	7	8	9
Age (years)	71	60	74	70	63	70	64	64	64
Sex	M	F	F	F	F	F	F	M	M
GOLD stage (1, 2, 3)	2	3	3	3	2	1	3	2	3
FEV_1_ (pp)	64.2	42.8	59.2	38.5	77.9	88.9	31.9		38.8
FVC (pp)	86.7	79.6	106.9	62.6	98.1	155.4	90.3		101.2
FEV_1_/FVC	56.88	45.22	45.86	50.85	65.57	47.24	29.68		38.3
Packs per year	52	44	40	86.75	47.75		27	92	50
Sustained smokers	No	No	No	Yes	No	No	No	No	Yes
Delay between symptoms and visit to the clinic	7	3	5	7	6	4	9	11	5

Individual data are presented for age, sex, FEV_1_, FVC, FEV_1_/FVC and packs per year for our COPD patients. F = female, M = male, COPD = chronic obstructive pulmonary disease, GOLD = Global Initiative for COPD; FEV_1_ = forced expiratory volume in 1 second; FVC = forced vital capacity; pp = percent predicted.

### Pyrosequencing outcomes

A total of 142,786 raw reads were obtained from 16S rRNA gene pyrosequencing 18 DNA sputum samples from the 9 COPD patients. P6 was excluded from the microbiota profiling analysis because of low sequences numbers (225 sequences), however qPCR analysis were still performed. A total of 68,400 sequences (3,800 sequences per sample) were available for analysis. Overall, after normalization, 2,267 OTUs were identified.

### Sputum diversity indices from COPD patients

**Fig 1:**
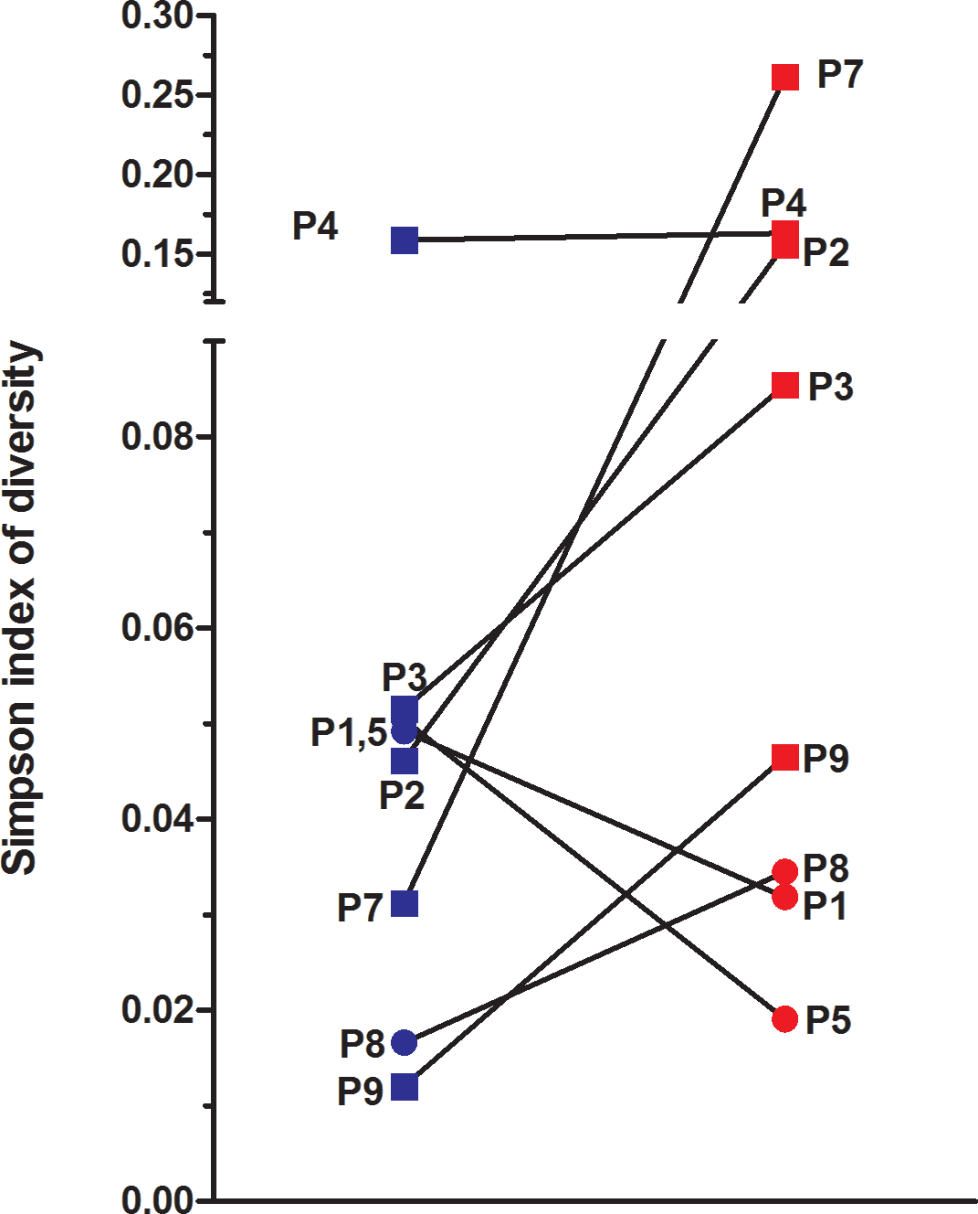
Patient-specific measures of Simpson diversity index in COPD sputum (OTU_0.97_ level). Simpson diversity index was calculated within mothur from sputum samples of stable (blue) and exacerbated (red) COPD patients. Gold 2 patients (circle) and GOLD 3 patients (square) are shown in the figure.

Alpha-diversity was represented using the Simpson’s diversity index (Fig 1). As shown in Fig 1, patient-specific variation in alpha-diversity nicely illustrates a difference from a clinically stable state versus an exacerbation. As an example, P7’s Simpson’s index had a one log increase from 0.031 to 0.26 during exacerbation.

### Phylum diversity in sputum of COPD patients

**Fig 2:**
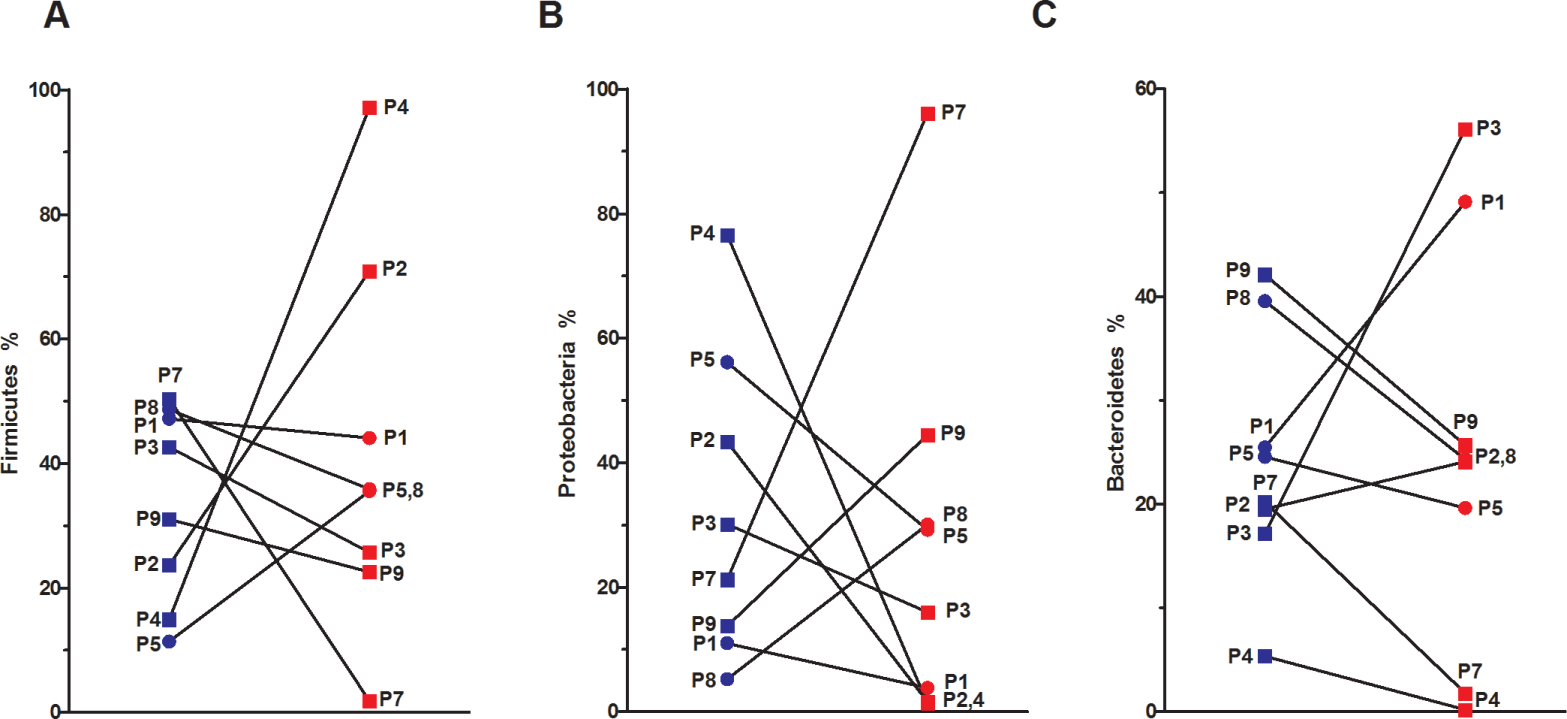
Sequence proportions of main phyla found in sputum samples of paired stable and exacerbated COPD patients. Sequence proportion of phyla were calculated from mothur. Patient-specific values of **(A)** *Firmicutes*, **(B)** *Proteobacteria* and **(C)** *Bacteroidetes* sequence proportion were analyzed from stable (blue) and exacerbated (red) COPD patients. GOLD classification is also provided: GOLD 2 (circle) and GOLD 3 (square).

At the phylum level, *Firmicutes*, *Proteobacteria* and *Bacteroidetes* were dominant during exacerbations with average proportions of 41%, 28%, and 25% per sample, respectively (data from our datasets, see Materials and methods for datasets info). *Actinobacteria* (3%), *Fusobacteria* (2%), *Tenericutes*, *Spirochaetes*, and unknowns (<1%) composed the remaining phyla found in the sputum samples. Slight but non statistically-significant phylum shifts were seen in the sputa of COPD patients during the exacerbation when averaged across all samples. However, when using the same approach as alpha-diversity, our paired-patient analysis showed heterogeneous shifts at the phylum level (Fig 2). As an example, the sputum of P4 showed an 82% increase in *Firmicutes* while there was a 48% decrease in the sputum of P7 during the exacerbation. P7 showed a clear shift from *Firmicutes* to *Proteobacteria* (75% increase, Fig 2B).

### Microbiota diversity at the genus level in COPD patients

**Fig 3:**
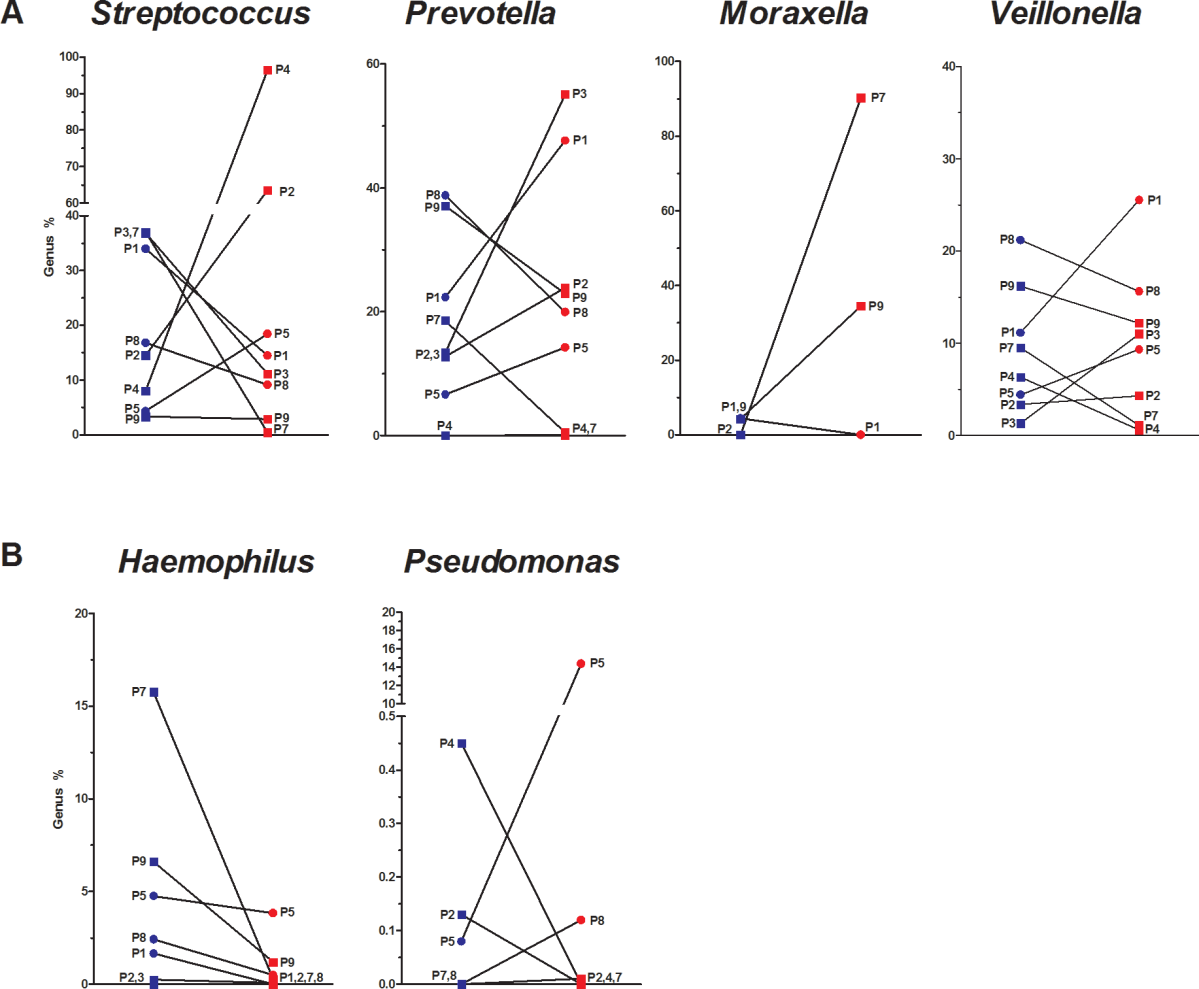
Patient-specific genera shifts in sputum samples during exacerbation. Sequence proportions of genera were calculated using mothur. Patient-specific values of **(A)** main and **(B)** clinically important genera sequence proportion found in sputum of stable (blue) and exacerbated (red) COPD patients. GOLD classification is also provided: GOLD 2 (circle) and GOLD 3 (square).

A total of 34 different genera were found in the sputa of COPD patients. Major genus proportional shifts were found during exacerbations compared to the stable states. Dominant and clinically-relevant genera are represented in Fig 3. The paired-patient analysis shows that *Streptococcus* (27%), *Prevotella* (23%), *Moraxella* (16%) and *Veillonella* (10%) were the dominant genera during the exacerbations (Fig 3A). The exacerbations showed an 88% increase in *Streptococcus* and a 90% increase in *Moraxella* in the sputum of P4 and P7, respectively. Clinically-important genera were also screened (Fig 3B). *Streptococcus* (27%) and *Moraxella* (16%), previously described, were the 2 main clinically-important genera found in the sputum of COPD patients during exacerbations. A low proportion of the microbiota detected during the exacerbations was composed of *Pseudomonas* (1.8%) and *Haemophilus* (0.7%).

### Bacterial and viral quantification in sputum of COPD patients

**Fig 4:**
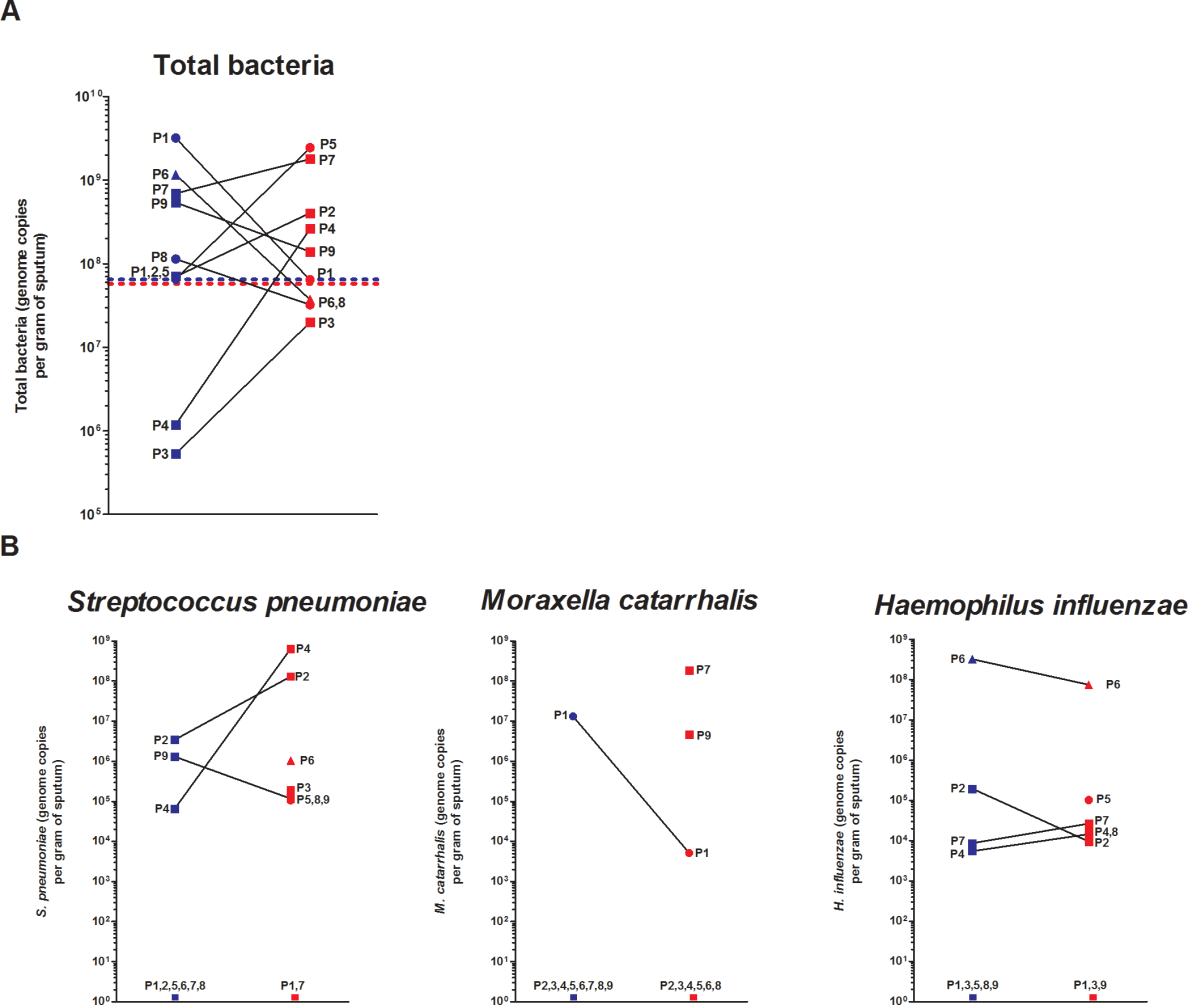
Comparisons of total bacterial load and select pathogens in sputum of stable and exacerbated COPD patients. Mean (dash lines) and patient-specific values of total bacteria **(A)** and patient-specific pulmonary pathogens **(B)** were detected from sputum samples of stable (blue) and exacerbated (red) COPD patients with qPCR. GOLD classification is also provided: GOLD 1 (triangle), GOLD 2 (circle) and GOLD 3 (square).

Mean total bacterial charge did not vary between stable (6.5×10^8^±1x10^9^) and exacerbated (5.8×10^8^±9x10^8^) states (Fig 4A). However P3 and P4 had a two log increase while P1 had a two log decrease in their total bacterial loads (Fig 4A). Clinically-important pathogens were also detected in high concentrations during exacerbations (Fig 4B). The exacerbations induce a 4 log increase in *S*. *pneumoniae* and an 8 log increase in *M*. *catarrhalis* in P4 and P7, respectively (Fig 4B). Our microbiota analysis did not suggest the importance of the genus *Haemophilus* and the same trend was observed between the qPCR and the microbiota analysis. However, *H*. *influenzae* was detected with qPCR in high concentrations in P6 (Fig 4B). *Pseudomonas aeruginosa* was not detected in any sputum samples with this specific set of primers during qPCR (detection limit = 10 copies/g of sputum), even if our microbiota analysis showed the presence of *Pseudomonas* (~15%) during exacerbation in the sputum of P5 (Fig 3B). This difference may come from the fact that it might be another pseudomonas species.

Out of the 6 targeted respiratory viruses with qPCR, only Influenza B was found in the sputum of P2 in high concentrations during exacerbation, with 3.4×10^7^ genome copies per gram of sputum. RSV A and B were detected in P7 and P4, respectively, with low genome copies per gram of sputum (1×10^3^, not shown). Influenza A, HRV and adenovirus were below detection limits (<10–100 genome copies/g of sputum) in all samples.

### Comparison of sputum microbiotas in GOLD 2 and GOLD 3 patients during exacerbations

**Fig 5:**
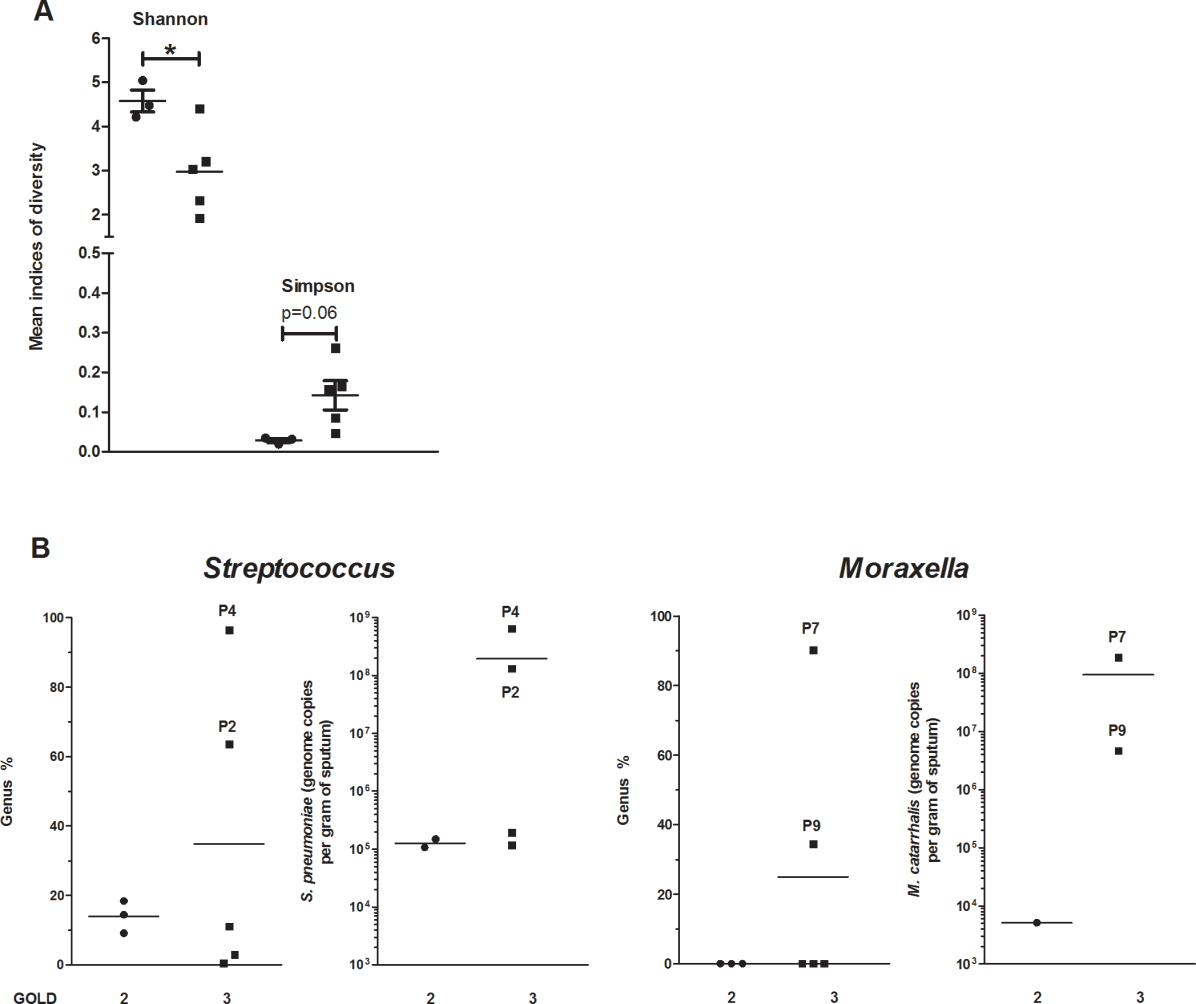
Clear differences in the pulmonary microbiota of Global Initiative for Chronic Obstructive Lung Disease (GOLD) 3 COPD patients compared to GOLD 2 COPD patients. Mean Shannon and Simpson diversity indices values **(A)** of GOLD 2 (black circle) and GOLD 3 patients (black square) were measured during exacerbation. Mean genus proportions and qPCR values of clinically important pathogens were quantified **(B)** in the sputum of GOLD 2 (black circle) and GOLD 3 (black square) COPD patients. *: p≤0.05.

As shown in Fig 5A, diversity indices in GOLD 3 COPD patients were different from those of GOLD 2 patients. Shannon index (a community richness index) was significantly lower and Simpson index (representing evenness) tended to be higher in GOLD 3 patients. These results highlight a shift in microbiotas of GOLD 3 patients compared to GOLD 2. *Firmicutes* and *Proteobacteria* were detected in GOLD 3 compared to GOLD 2 COPD patients during exacerbations, while *Bacteroidetes* seemed to be lowered (data from our datasets, see Materials and methods for datasets info). Two clinically important pathogens were detected in GOLD 3 compared to GOLD 2 patients during exacerbations (Fig 5B). GOLD 3 patients P2 and P4 had increases in *S*. *pneumoniae* while P7 and P9 had increases in *M*. *catarrhalis* (Fig 5B).

## Discussion

In this study, we evaluated the sputa microbiota of patients with COPD during stable conditions and subsequently when they were exacerbated.

### Culture-independent detection of respiratory pathogens in COPD sputa

In our study, culture-independent methods were used and showed no difference in mean total bacterial loads in the sputa of COPD patients between stable conditions and during AECOPDs. Our data are, however, within the range of total bacteria in sputum [38]. In contrast, Garcha *et al*. showed an increased bacterial load in the sputum of exacerbated COPD patients compared to stable conditions [19]. The difference with our study could be related to the small number of samples and to heterogeneity in the behavior of bacterial loads during an AECOPD from one patient to another. For example, P4 had a 2 log increase in total bacterial load during the exacerbation period. Therefore, the finding that the mean total bacterial load did not change during an AECOPD should be interpreted with caution.

*S*. *pneumoniae*, *M*. *catarrhalis* and *H*. *influenzae* are known respiratory pathogens linked to COPD exacerbations and they were detected in high concentrations in the sputa of our exacerbated COPD patients (up to 8 log increase compared to a stable state). These results emphasize that these specific respiratory pathogens were likely to have triggered the exacerbation of our COPD patients. *P*. *aeruginosa* was not detected in our study, probably because we did not enroll patients with very severe COPD [39]. Among all genera revealed with the pyrosequencing of 16S rRNA gene (E.g. *Streptococcus*), significant concentration of specific species were detected with qPCR (E.g. *S*. *pneumoniae*). These specie specific qPCRs data might not represent the whole population (E.g. streptococci) found in the microbiota data. Nevertheless, our qPCRs data seems to correlate (E.g. P4) with the microbiota data obtained.

### Viral characterization during exacerbation in COPD sputum

The importance of viruses in COPD exacerbation has been previously underestimated, but several studies have recently highlighted their contributions [40–43]. Overall, key pulmonary viruses were mostly absent in the sputum of our COPD patients even if Influenza B was detected in one exacerbated patient. In a future study, random viral sequencing will be used instead of focusing on specific viruses. On average, patients waited six days following the onset of symptoms before visiting the clinic. This could have reduced the likelihood of identifying viruses in our specimens. The Influenza B positive patient was seen at the COPD clinic within three days of symptom onset, the recommended delay, suggesting that a shorter period between onset of symptoms and sputum collection might increase the odds of identifying viruses during AECOPD.

### Sputum microbiota during AECOPD

COPD is a heterogeneous disease and this is further highlighted by the sputa microbiota at baseline and during AECOPD (Fig 2). As a result, it was not possible to draw a unique conclusion regarding the behavior of the sputa microbiotas during AECOPD. Nevertheless, using a paired-patient analyses, the identity of which microorganism may be responsible for that exacerbation in our COPD cohort could be established. However, we are aware that we cannot rule out the possibility that these shifts may be the results of normal temporal dynamics.

Three out of nine patients (P2, 4 and 5) had increases in Gram-positive bacteria (*Firmicutes*, which includes *Streptococcus*, *Staphylococcus* and *Veillonella*) during exacerbations, while three others (P7, 8 and 9) had their sputum enriched in Gram-negative bacteria (*Proteobacteria*, which includes *Moraxella*, *Haemophilus* and *Pseudomonas*). These population changes have also been shown in other studies for these same two phyla [2, 15, 27]. Two patients out of nine (P1 and 3) showed a different microbiota shift involving increases in *Bacteroidetes* (which includes *Prevotella*). At the genus level, P1 and P3 had an increase in *Prevotella* relative abundances during exacerbations. *Prevotella*, a Gram-negative anaerobic bacteria, has never before been linked to exacerbated episodes, even though it has already been linked to chronic bronchitis [44]. Perhaps, anaerobic bacteria may be underestimated in the onset of COPD exacerbations. Overall, microbiota shifts were seen in all COPD patients during exacerbation and interestingly our specific pulmonary pathogens detected with qPCRs correlate nicely with the microbiota findings.

### Clear microbiota shifts in the sputa of GOLD 3 COPD patients

Studying the sputum microbiotas and confirming the results with qPCR, microorganisms that were likely responsible for the AECOPDs were identified. In fact, the pathogens detected in high concentrations in the sputa during AECOPDs were either bacterial (*S*. *pneumoniae* (P2,P4) or *M*. *catarrhalis* (P7,P9)) or viral (Influenza B (P6, GOLD 1)). Interestingly, four of these patients are classified as GOLD 3 (P2, 3, 4, 7 and 9). Shannon diversity index decreased in the sputa of GOLD 3 patients during exacerbation compared to GOLD 2 patients (P1, 5 and 8; Fig 5A). Perhaps additional studies are required to clearly evaluate the impact of disease severity on the microbiota. Severe COPD state could predispose patients to a more pathogenic microbiota instead of the actual microbiota inducing disease severity.

### Study limitations

One limitation of this pilot study is the small number of evaluated patients, a critique that also applies to other studies on the same topic [45]. However, this limitation was somewhat mitigated by a strong study design involving pair-wise comparisons, enabling us to study potential shifts in the sputa microbiotas of each individual patient. As previously mentioned, COPD patients waited, on average, six days before visiting the COPD clinic. Shortening this delay may result in better viral characterization during exacerbations.

## Conclusions

Little is known about the comparative microbiotas of stable and exacerbated COPD patients using a non-invasive method. Our study shows that the sputum microbiotas of COPD patients is complex due to the heterogeneous nature of COPD and the small number of evaluated patients in our study, it is not possible to make general treatment recommendations for AECOPD. Nevertheless, our data contributes to a better understanding of which microorganisms could be induced during exacerbations. As stated before, we cannot rule out the possibility of random fluctuations over time. Our study, however, clearly shows dramatic microbiota shifts occurring during AECOPD, mostly as it relates to *Firmicutes* or *Proteobacteria*. However, the nature of these shifts varies from patients to patients in such a way that the treatment should be patient-specific. Additional studies are required to further investigate the impact of microbial diversity in COPD patients.

## Supporting information

S1 Table Primers and probes. List of all primers and probes used for qPCR analysis.
(DOCX)
