# Supplementary material for: Exacerbation induces a microbiota shift in sputa of COPD patients
Source: PLoS One. 2018 Mar 26;13(3):e0194355. doi: 10.1371/journal.pone.0194355 (PMC5868791; doi:10.1371/journal.pone.0194355)
Supplement: S1 Table — List of all primers and probes used for qPCR analysis. (DOCX) [file pone.0194355.s001.docx]

**S1 Table. List of primers and probes.**

| Microorganism | Target | Primers and probes | Sequences |
| --- | --- | --- | --- |
| Bacteria |  |  |  |
| *P. aeruginosa* | *oprL* gene | PAO1-F  PAO1-R  PAO1-TM | CAG GTC GGA GCT GTC GTA CTC  ACC CGA ACG CAG GCT ATG  FAM-AGA AGG TGG TGA TCG CAC GCA GA-BHQ1 |
| *H. influenzae* | *P4* Lipoprotein gene | Hin-F  Hin-R  Hin-TM | CCG GGT GCG GTA GAA TTT AAT AA  CTG ATT TTT CAG TGC TGT CTT TGC  FAM-ACA GCC ACA ACG GTA AAG TGT TCT ACG-BHQ1 |
| *M. catarrhalis* | *CopB* gene | Mor-F  Mor-R  Mor-TM | GTG AGT GCC GCT TTT ACA ACC  TGT ATC GCC TGC CAA GAC AA  FAM-TGC TTT TGC AGC TGT TAG CCA GCC TAA-BHQ1 |
| *S. pneumoniae* | *Spn9082* (gene fragment) | Spn-F  Spn-R  Spn-TM | AGT CGT TCC AAG GTA ACA AGT CT  ACC AAC TCG ACC ACC TCT TT  FAM-TAC ATG TAG GAA ACT ATT TTC CTC ACA AA-BHQ1 |
| Viruses | target | Primers and probes | Sequences |
| MS2 phage | Assembly/Coat protein | MS2 1-for  MS2 1-rev  MS2 1-probe | GTC CAT ACC TTA GAT GCG TTA GC  CCG TTA GCG AAG TTG CTT GG  FAM-ACG TCG CCA GTT CCG CCA TTG TCG-BHQ1 |
| Influenza A | M fragment | InfAfor  InfArev  InfAprobe | GAC CRA TCC TGT CAC CTC TGA  AGG GCA TTY TGG ACA AAK CGT CTA  FAM-TGC AGT CCT CGC TCA CTG GGC ACG-BHQ1 |
| Influenza B | N/A | InfBfor  InfBrev  InfBprobe | TCC TCA ACT CAC TCT TCG AGC G  CGG TGC TCT TGA CCA AAT TGG  FAM-CC AAT TCG A/ZEN/G CAG CTG AAA CTG CGG TG-BHQ1 |
| RSV A | Nucleocapsid | RVSA-F  RSVA-R  RSVA-TM | GCT CTT AGC AAA GTC AAG TTG AAT GA  GCC ACA TAA CTT ATT GAT GTG TTT CTG  FAM-ACA CTC AAC AAA GAT CAA CTT CTG TCA TCC AGC-BHQ1 |
| RSV B | Nucleocapsid | RVSB-F  RSVB-R  RSVB-TM | GAT GGC TCT TAG CAA AGT CAA GTT AA  TGT CAA TAT TAT CTC CTG TAC TAC GTT GAA  FAM-TGA TAC ATT AAA TAA GGA TCA GCT GCT GTC ATC CA-BHQ1 |
| Rhinovirus | 5' untranslated region | HRV-F  HRV-R  HRV-TM | GTG AAG AGC CSC RTG TGC T  GCT SCA GGG TTA AGG TTA GCC  FAM-TGA GTC CTC CGG CCC CTG AAT G-BHQ1 |
| Adenovirus | Hexon | ADN-F  ADN-R  ADN-TM | GCC CCA GTG GTC TTA CAT GCA CAT C  GCC ACG GTG GGG TTT CTA AAC TT  FAM-TGC ACC AGA CCC GGG CTC AGG TAC TCC GA-BHQ1 |

*Definition of abbreviations:* N/A = not applicable.
